# Influence of a Modified Procedure of Joining Ceramic Head and Adapter Sleeve on the Stem Taper in Revision: An Experimental Study

**DOI:** 10.3390/bioengineering11020170

**Published:** 2024-02-09

**Authors:** Sandra Hunger, Christian Rotsch, Florian Günther, Welf-Guntram Drossel, Christoph-Eckhard Heyde

**Affiliations:** 1 Fraunhofer Institute for Machine Tools and Forming Technology IWU, 01187 Dresden, Germany; christian.rotsch@iwu.fraunhofer.de (C.R.); florian.guenther@iwu.fraunhofer.de (F.G.); welf-guntram.drossel@iwu.fraunhofer.de (W.-G.D.); 2 Department of Orthopaedic, Trauma and Plastic Surgery Clinic, University of Leipzig Medical Center, 04103 Leipzig, Germany; christoph-eckhard.heyde@medizin.uni-leipzig.de; 3 Institute for Machine Tools and Production Processes, Faculty of Mechanical Engineering, Chemnitz University of Technology, 09111 Chemnitz, Germany

**Keywords:** adapter sleeve, ceramic head, insertion, modular hip implant, revision surgery, surgical procedure, total hip arthroplasty

## Abstract

In revision operations, ceramic heads of modular hip implants can be replaced. As the surface of the stem taper can be damaged, additional adapter sleeves are applied. The components are usually connected manually by the surgeon in a one-step procedure by hammer impacts. In this study, we investigated a two-step joining procedure with reproducible impaction force. First, the adapter sleeve and head were joined quasi-statically with a force of 2 kN using an assembly device. In the second step, these components were applied to the stem taper using a pulse-controlled instrument. For reference, the joints were assembled according to standard conditions using a tensile testing machine. An average pull-off force of 1309 ± 201 N was achieved for the components joined by the instrument, and the average measured values for the components joined by the testing machine were 1290 ± 140 N. All specimens achieved a force >350 N when released and therefore met the acceptance criterion defined for this study. This study showed that a modified procedure in two steps with a defined force has a positive effect on the reproducibility of the measured joining forces compared to previous studies.

## 1. Introduction

Modular implants are increasingly being used in hip revision arthroplasty [1,2], as it is possible to replace worn or damaged implant components without having to remove the ingrown components in the bone. This protects existing bone tissue and reduces operation time. The ceramic inlay, which has already been analyzed in a previous study [3], and the ceramic head can be replaced. Manufacturers of hip implants recommend the use of metallic adapter sleeves for the revision of ceramic heads, as damaged surfaces on the stem taper can have a significant impact on the life span in situ of the implant [4]. Adapter sleeves are also used for lengthening corrections or complications due to hip prosthesis dislocations [5].

Damage to the stem taper may occur due to wear during use or removal of the old head. Surface damage or abrasion particles can cause punctual loads and the ceramic to break [6]. Despite this disadvantage, ceramic hip implant components offer clear advantages, such as low wear compared to metal-on-polyethylene joint pairings and longer life span in situ [7,8]. Using metal adapter sleeves creates new optimal conditions for connecting the ceramic head and hip stem. The three components, the head, adapter sleeve and femoral stem are connected by a radial press fit. Current instructions from manufacturers [4] recommend that the adapter sleeve should be placed first on the stem and connected to the taper with light pressure and a half turn. Next, the ceramic head is placed on top and joined with a hammer and one or several hammer impacts. The force for joining the three components is thus applied in a single-step process. The direction and amount of the applied force determine the connection stability [9,10,11,12]. The force applied varies depending on the surgeon [13]. In a study involving 39 surgeons from German clinics, Nassutt et al. [14] analyzed the axial taper forces of the ceramic head and hip stem connection in comparable situations. The forces varied between 273 N and 7848 N; the authors concluded that there is an urgent need to make the insertion method reproducible. Insufficient joining forces lead to inadequate joining connections, leading to premature loosening and micro-movement-induced corrosion or abrasion [10]. Metal abrasion at the junctions of the modular implants is assumed to be a reason for the failure of total hip replacements [15,16]. Forces that are too high pose a risk of fractures of the surrounding bone [17] or breakage of ceramic implant components.

Several studies showed that the joining force has the most significant influence on the taper connection, followed by other factors such as the contact situation (e.g., contamination such as fat [18]), the head material [11,19] or the design of the implant [20]. However, the most significant factors influencing the assurance of a secure taper connection are the joining process and the surgical technique [21,22].

Optimal and consistent conditions during the joining of hip implant components can minimize risks such as ceramic fractures, metal abrasion at the taper connection or fractures of the surrounding bone. This can lead to an increase in the life span in situ of the implant. The new modified procedure of joining investigated could contribute to the standardization of the surgical technique, which makes the joining force applied independent of the surgeon, reduces or saves costs for follow-up operations and has a positive effect on the patient’s quality of life. This experimental study under laboratory conditions aimed to analyze the influence of a modified joining procedure on the joint strength of the ceramic head, adapter sleeve and hip stem. The question was how reproducible joining in two individual steps with a constant joining force affects the press-fit connections. A new process was developed, which connected the adapter sleeve to the ceramic head in the first step and these two components to the stem taper in the second step. The combination of an instrument and a new assembly device applied a reproducible joining force of 2 kN in both the process steps. The pull-off forces of these connections were compared with the pull-off forces of the components that were joined under optimal conditions using a testing machine. Furthermore, the measurement deviations of the forces were compared with those from previous studies in the literature.

## 2. Materials and Methods

**Figure 1:**
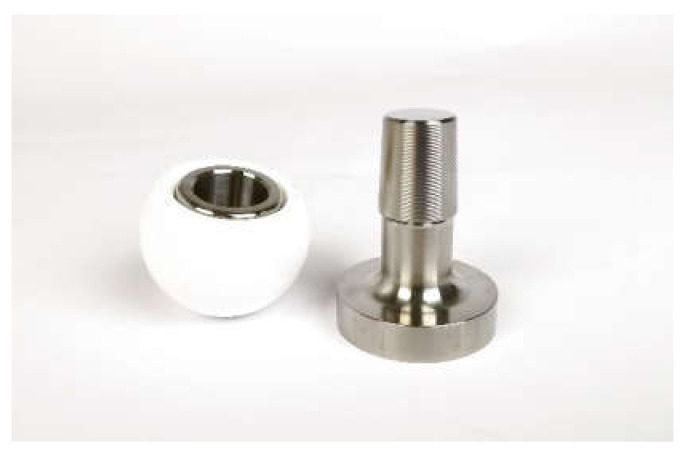
Head Taper Components Used for the Tests.

The investigations were carried out as part of a BMBF project called “Smart-I” together with the implant and instrument manufacturers (see the acknowledgments). The participating companies provided both the CAD data and the test components. A 32 mm ceramys revision head from Mathys Orthopädie GmbH (Mörsdorf, Germany) and a BioBall adapter sleeve 12/14 standard, neck length L from Merete GmbH (Berlin, Germany) were examined. These were combined with a 12/14 stem taper, which was specially adapted and manufactured for the project by Aristotech Industries GmbH (Luckenwalde, Germany). The part of the femoral stem located in the bone was removed to simplify the mounts for the experiments (see Figure 1). The revision head was made of alumina-toughened zirconia (ceramys^®^, Mathys Orthopädie GmbH, Mörsdorf, Germany), and the taper and the BioBall adapter sleeve were made of Ti6Al4V.

### 2.1. Concept of the Two-Step Joining Procedure

The new concept to be investigated was that the head, adapter sleeve and stem taper joining process should be carried out in two separate process steps. The same reproducible joining force was applied in both steps. First, the adapter sleeve was pressed into the head and in the second step, these two components were joined together on the stem taper.

**Figure 2:**
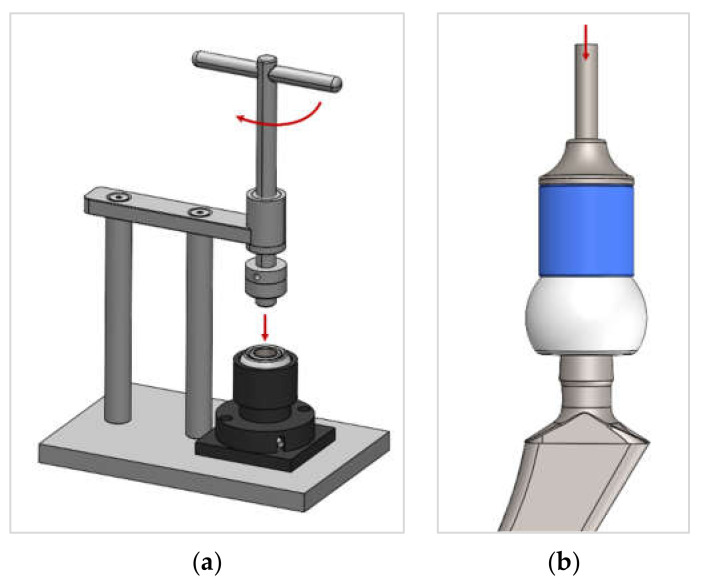
Concept of the two-step joining process: 1. Joining of adapter sleeve and head with assembly device (**a**) and 2. Joining of adapter sleeve/head and stem taper with instrument (**b**).

To connect the adapter sleeve and head, an existing assembly device from Mathys [23] was adapted to press the sleeve into the head with 2 kN (see Figure 2a—CAD planning of the first joining step). Optimal, straight alignment during the joining process was achieved by inserting the head into a holder adapted to the outer contour, which allowed small, self-aligning movements of the head in the holder. An integrated O-ring held the head in the holder, which allowed compensating movements. When the lever of the device was turned, the axle moved downwards. At the end of this axle was a counterpart that was precisely adapted to the inner contour of the adapter sleeve so the ball could be self-centered and axially aligned. The force was applied to the upper edge of the adapter sleeve by manually turning the lever until the target force of 2 kN was reached.

The ceramic head and the revision adapter sleeve were joined to the taper of the hip stem using an instrument designed by Endocon GmbH (Wiesenbach, Germany), which applied a reproducible, pulse-controlled joining force and was already available on the market under the brand name safeConnect^®^ [24] (see Figure 2b). The impulse of the instrument was adjusted to a maximum force of 2 kN for these tests to comply with the force specified in the ISO 7206-10 test standard [25]. In this step, the pre-assembled components, adapter sleeve and ceramic head were placed manually on the taper of the hip stem. The instrument was then placed axially on the top of the head and the impulse was triggered by pressing on the instrument, which connected the components with each other through the impulse controlled force.

### 2.2. Impacting and Pull-Off Tests

**Figure 3:**
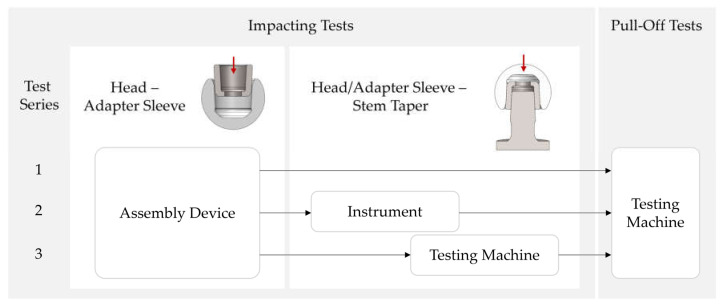
Experimental design.

In order to test the concept of joining in two steps with reproducible force application, the following test plan (see Figure 3) was worked out:

- Test series 1: Joining of adapter sleeve in head with the assembly device and pull-off tests with the testing machine;
- Test series 2: Joining of adapter sleeve and head with assembly device and manual impacting of these two components with the instrument onto the stem taper; pull-off tests with the testing machine;
- Test series 3: Joining of adapter sleeve and head with assembly device and impacting of these two components onto the stem taper with the testing machine according to ISO 7206-10 [25]; pull-off tests with the testing machine.

To evaluate the reproducibility of the new joining concept, a comparison of the pull-off forces of test series 2 and 3 was carried out. A *t*-test of the two samples was performed to evaluate the significance. The aim of test series 1 was to find out which of the two press-fit connections was the first to loosen during the pull-off tests.

A new set of components was available for each test series. Due to the expected plastic deformation on the press-fit surfaces during the first joining process [26], the first test was excluded from the evaluation. The components were cleaned of possible abrasion residues and dried between the individual tests. All components were positioned by hand, as straight as possible and without pressure, before the impaction force was applied using the assembly device, instrument or testing machine.

**Figure 4:**
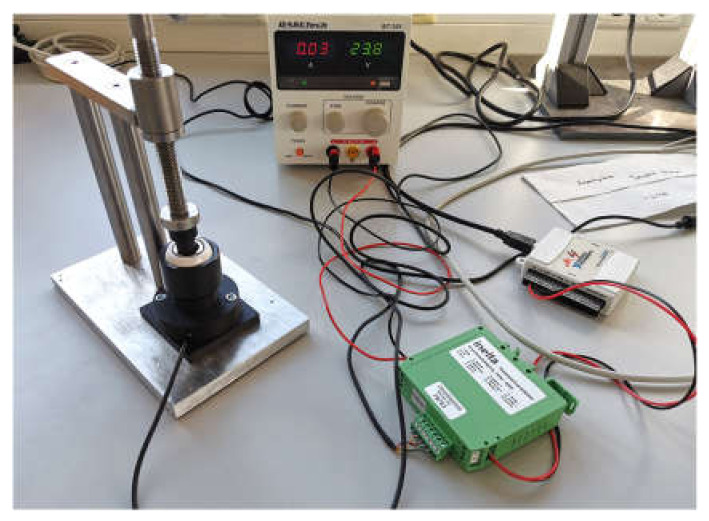
Test setup 1 for joining adapter sleeve in ceramic head.

To realize the joining process of head and adapter sleeve reproducibly with 2 kN, a sensor was added to the assembly device from Mathys. In accordance with the desired measuring range, a load cell with ball support (KMM60-10 kN) including measuring amplifier (IMA2-DMS) from Inelta Sensorsysteme GmbH & Co. KG (Taufkirchen, Germany) was mounted below the ceramic head holder. The sensor data was read out via a multifunctional DAQ device (USB-6009, National Instruments Corporation, Austin, TX, USA), which additionally served as an interface to the PC (see Figure 4). Finally, the measured values were visualized using a specially developed LabVIEW code (National Instruments Corporation, Austin, TX, USA) to terminate the manual joining process when the target force was reached.

**Figure 5:**
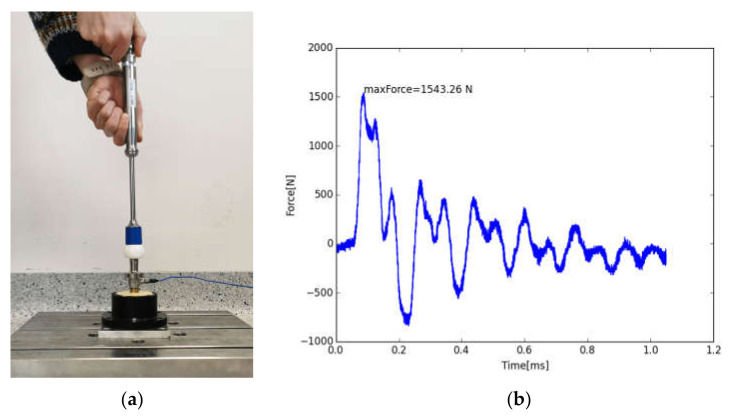
Test setup 2 (**a**) and exemplary force–time curve of the joining process (**b**). The joining impulse is followed by decaying vibrations and smaller impulses due to the springback of the instrument.

When joining with the instrument (test series 2), the force was transferred to the components via a pulse triggered in the handpiece. For this purpose, the stem taper was connected to a force transducer via a thread. With this piezoelectric force transducer, a force–time curve could be recorded to determine the maximum force (see Figure 5). The assembly was embedded in a fixture that stood straight on the floor via a plate to exclude damping from underground. The test setup and an exemplary force–time curve for the joining process can be seen in Figure 5.

**Figure 6:**
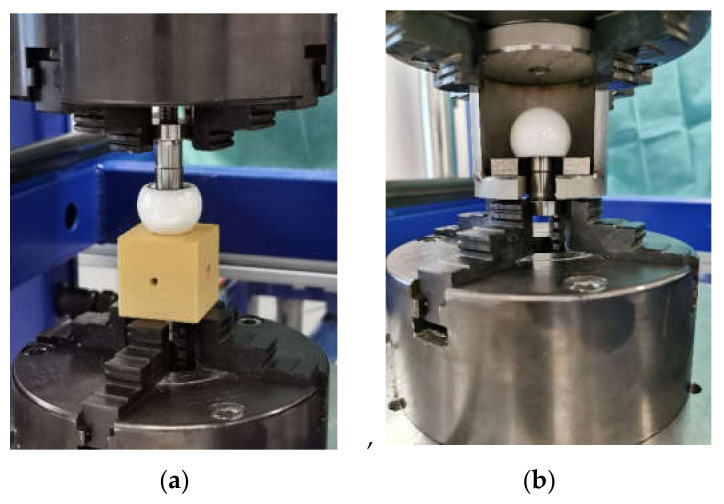
Impacting (**a**) and pull-off (**b**) with the testing machine.

A tensile/compression/torsion testing machine from DYNA-MESS Prüfsysteme GmbH, Aachen, Germany, was used to join the assembly ceramic head/adapter sleeve and the stem taper in test series 3 and to pull off all the assemblies. Impacting and pull-off tests were carried out in accordance with ISO 7206-10 [25] at a test speed of 0.04 mm/s. Figure 6 shows the test setups for impacting and pull-off.

The results were statistically analyzed and graphically displayed using OriginPro 2023 software (OriginLab, Northampton, MA, USA). To evaluate the influence of the new joining procedure, a two-sample independent *t*-test (hypothesis test) was used to determine whether the mean values of our two samples differed significantly from each other. The significance level was α=0.05.

## 3. Results

For the insertion of the adapter sleeve into the head with the modified assembly device, the mean value was 2030 N with a standard deviation of approx. 22 N. The mean value for the pull-off test was 1546 N, with a standard deviation of 77 N. No outliers were detected. The measured values are listed in Table 1.

The measured values of test series 2 and 3 are summarized in Table 2. The mean value for joining the adapter sleeve into the head from test series 2 was approx. 2146 N and for test series 3 approx. 2026 N, whereby the standard deviation of test series 3 for the testing machine was lower. This was because the fifth test in series 2 was an outlier. The average impaction forces connecting the head/adapter sleeve and the stem taper were approx. 1581 N for the instrument (test series 2). This resulted in a standard deviation of approx. 298 N. In contrast, the test machine could join with an impacting force of 2002 N and the standard deviation was only 1.4 N (test series 3). When removing the head from the taper stem, an average pull-off force of approx. 1309 N was achieved for the components joined by the instrument and the average measured values for the components joined by the testing machine were 1290 N. The standard deviation for the pull-off forces for the instrument was approx. 202 N and for the testing machine lower at approx. 140 N. In all the tests, the adapter sleeve and head always detached together from the stem taper.

Although the measured difference between the impacting and pull-off forces of the head/adapter sleeve on the taper stem was higher for the instrument than for the testing machine, the release forces of the tests with the instrument were higher on average than with the testing machine. However, they only differed by approx. 19 N, which was within the standard deviation determined. The difference between the two average pull-off forces of test series 2 and 3 was not significant for a significance level of 0.05 and a *p*-value of 0.87 (t(7.14)=0.17).

**Figure 7:**
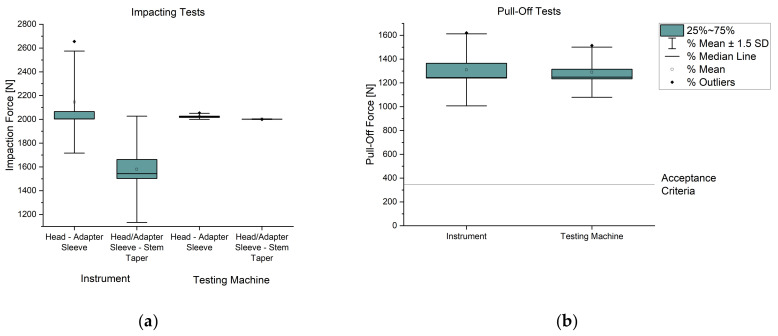
Boxplot diagrams for the impacting (**a**) and pull-off tests (**b**).

The ISO 7206-10 standard [25] does not specify any minimum requirements for pull-off forces. Therefore, the internal acceptance criterion for the pull-off forces was set at 350 N in the project. This requirement was met for all the tests. The results are shown below in two boxplot diagrams (see Figure 7).

## 4. Discussion

In several studies in surgical treatment with total hip endoprostheses, the use of devices is described as necessary for a more reproducible joining process [11,12,20], as the long-term success of such an operation primarily depends on the surgical technique. One parameter that significantly influences the quality of the connection is the impaction force [10,27,28].

Ceramic–ceramic friction partners are often used in total hip arthroplasty, as they generate minor abrasion and therefore have a longer service life. However, the ceramic components place high demands on the joining process to prevent ceramic fracture. For revision operations, adapter sleeves are used to create a new, smooth surface between the ceramic head and stem taper, thus preventing stress peaks caused by abrasion particles. This creates a second joining connection on the modular implant, which offers additional potential for errors. Current handling instructions and the instruments mainly used leave plenty of scope for variations in the impaction force applied to connect the three implant components.

To make the joining process of a ceramic head reproducible during a revision operation using an adapter sleeve, our study investigated a modified procedure in two process steps with a constant impaction force. In the first step, the adapter sleeve was joined to the ceramic head with 2 kN and in the second step, these two components were pressed on the taper of the hip stem with an instrument that generated a reproducible force pulse of 2 kN.

By following this new joining procedure, the adapter sleeve and head detached from the stem taper first in all the pull-off tests. The connection between the adapter sleeve and head remained intact. The pull-off forces $F_{POF_{1}}$ of the adapter sleeve and head from test series 1 also confirmed this, which at an average of 1546 N were higher than the pull-off forces $F_{POF_{2}}$ and $F_{POF_{3}}$ from test series 2 and 3. When joining the adapter sleeve to the head, there was an upward outlier (test no. 5 on the instrument) caused by inattention. The measured impaction force of the head/adapter sleeve on the stem taper was also higher than the previous tests. However, this was not reflected in the pull-off force, so this could be neglected. An additional mechanical impaction force limitation, like a stopper on the assembly device, would make this joining step more reproducible and reduce the possibility of outliers.

The release forces $F_{POF_{2}}$ of the components joined with the instrument were approximately the same as for the test components assembled with the testing machine ($∆F_{POF}\approx 10\;N$). However, the measured impacting forces $F_{I{FI}_{2}}$ on the instrument did not reach 2 kN (they were on average 1580.5 N). It is assumed that this was due to the damping behavior of the test setup (the environment) [14,29]. As the instrument was a product available on the market and calibrated by Endocon GmbH, it was expected that the instrument applied the joining force of 2 kN. The results of the pull-off forces seemed to confirm this assumption. The standard deviation of the impaction forces was significantly higher for the instrument at 298 N than for the testing machine at 1.4 N (∆SD≈297 N). This was an expected result, as the testing machine was regulated to a target force of 2 kN. In a study by Nassutt et al. [14], the mean measured forces during joining by different test persons were 2927 ±2059 N. Wendler et al. [13] determined forces of 2037.2 N ± 724.9 N. These studies show that the standard deviation for the impaction forces on conventional joining methods is significantly higher than in our tests with the instrument and the previously joined head/adapter sleeve assembly. The surgeons in the study by Nassutt et al. carried out 3–5 tests in their own test series and the standard deviation varied between 21 and 1414 N. This indicates that some surgeons generate repetitive reproducible joining forces while others have a more significant variation. For inexperienced surgeons in particular, the new workflow with assembly device and instrument could generate consistent impacting forces and thus mean a more reproducible application of force. The study also described that some surgeons were reluctant to hammer on the ceramic head. This is why joining forces below 2 kN were measured. With such low forces, the risk of metal abrasion or micro-movement-induced corrosion at the taper connection is higher than with greater impaction forces, which the new, reproducible joining procedure could prevent.

All joined connections achieved a pull-off force >350 N and therefore fulfilled the acceptance criterion in our study. The test series’ measured pull-off forces confirmed previous studies on ceramic revision heads using adapter sleeves [30]. With the standard deviations of the pull-off forces $F_{POF_{2}}$ and $F_{POF_{3}}$ of the instrument and testing machine, there was only a difference of approx. 60 N. The mean values of the two test series did not differ significantly (hypothesis test), which showed that the new joining procedure could withstand joining under almost optimal conditions such as the testing machine. As there is a linear relationship between the impaction force and the pull-off force for conical press-fit connections [10], it is reasonable to assume that the pull-off force is reproducible if the impaction force is applied at a constant level. However, this should be confirmed by further investigations with different applicants, as no data were found in the literature that would allow a comparison with our recorded data (same joining force of 2 kN, use of a ceramic head, evaluation of the standard deviation).

In this study, the influence of the angled positioning of the head/adapter sleeve connection on the stem taper was not an object of investigation. Attention was paid to ensuring that the alignment was as straight as possible by one test person and that the connection surfaces were dry and free of particles. One study shows that the best joint quality depends, among other factors, on an ideally straight alignment of the joining components to each other [21]. In their study, Ouellette et al. [29] described that the head taper connection aligns itself during insertion. To evaluate the influence of the modified joining procedure, tests should be carried out with different test persons under more realistic conditions. It must be verified whether an ideally straight load application during an operation is always feasible due to the accessibility. Other questions are at what angle the impulse of the instrument can still be triggered, how high the force losses are, and what influence this has on the connection quality. The impaction forces are divided into horizontal and vertical components with angular misalignment so that the resulting force becomes smaller the larger the angle of entry. Furthermore, some studies describe a force of 4 kN as the optimum impaction force for head taper [10,28,29,31,32], although the ISO 7206-10 [25] recommends a force of 2 kN. Various conditions influence the forces applied to the joint connection, such as the stiffness of the impact tip of the instrument [29] or the implant component size [33], so further investigations and detailed dimensioning are required to define the optimum joining force. Compensation could be achieved by using different tips or an adjustable force range of the instrument.

Another limitation of this study is that the pull-off forces of impulse-joined components were compared with quasi-statically joined parts. Wade et al. [21] concluded that the connection quality of quasi-statically joined components is the best, so a comparison with almost optimally quasi-statically joined parts using a testing machine seemed reasonable in our case. However, an impulse-joined test setup [31] would be preferable for more precise comparability of the results for further investigations. The influence of the environment on the connection quality as an attenuating component was also not investigated in detail in this study. Some studies on this have come to different conclusions [13,31]. The number of test samples available for the experiments was limited. A larger number would have provided further statistical support. Furthermore, only one head, adapter sleeve and stem taper size were analyzed, and the tests were carried out under simplified conditions in the laboratory.

## 5. Conclusions

The experiments in this study showed that a modified procedure for joining the ceramic ball, adapter sleeve and stem taper in two steps with a constant force positively affects the reproducibility of the measured impacting forces compared to previous studies. As there is a linear relationship between joining force and release force, it is assumed that this also increases the quality of the joint connection of modular hip implants. Further investigations under real operating conditions are still required to be able to evaluate this thesis conclusively. An extensive, systematic study of the various factors influencing the resulting force in the connection, such as the instrument’s alignment, damping behavior of the surrounding tissue, implant sizes or surgical access routes would be desirable to increase patient safety. A comparison with the currently used conventional joining process with several surgeons is also outstanding.

## Figures and Tables

**Table 1:** Impaction and pull-off force for test series 1.

Trial Number	Impaction Force Adapter $F_{IF_{1}}$ [N]	Pull-Off Force $F_{POF_{1}}$ [N]
1	2012	1468
2	2061	1463
3	2010	1576
4	2045	1636
5	2022	1588
Mean Value	2030	1546.2
Standard Deviation	22.2	77.3

**Table 2:** Forces for test series 2 und 3.

	Instrument (Test Series 2)			Testing Machine (Test Series 3)		
Trial Number	Impaction Force Adapter $F_{IF_{2}}$ [N]	Impaction Force Head/Taper $F_{I{FI}_{2}}$ [N]	Pull-Off Force $F_{POF_{2}}$ [N]	Impaction Force Adapter $F_{IF_{3}}$ [N]	Impaction Force Head/Taper $F_{IFTM_{3}}$ [N]	Pull-Off Force $F_{POF_{3}}$ [N]
1	2001	1663	1620	2017	2002	1514
2	2004	1503	1241	2027	2002	1234
3	2066	1184	1076	2054	2000	1249
4	2003	1543	1245	2010	2002	1315
5	2655	2009	1365	2020	2003	1138
Mean Value	2145.8	1580.5	1309.4	2025.6	2001.9	1290.2
Standard Deviation	286.0	298.0	201.8	17.0	1.4	140.4

## Data Availability

The data presented in this study are available on request from the corresponding author.
